# Effects of different nose types on class II treatments for female patients

**DOI:** 10.1186/s40510-019-0296-7

**Published:** 2019-12-02

**Authors:** Ozkan Semih Cankaya, Fatih Celebi, Ali Altug Bicakci

**Affiliations:** 1 Private practice, Istanbul, Turkey; 2 Department of Orthodontics, Faculty of Dentistry, Gaziosmanpasa University, 60100 Tokat, Turkey

**Keywords:** Camouflage treatment, Class II, Orthognathic surgery, Rhinoplasty

## Abstract

**Background:**

The aim of this study was to evaluate the effect of different nose types on the perception of facial aesthetics following camouflage treatment and orthognathic surgery for skeletal class II female patients.

**Methods:**

A pre-treatment profile photograph of a skeletal class II adult patient was selected from the department archive. Two constructed photographs were created to represent orthognathic surgery and camouflage treatments with the aid of computer software. A total of 18 constructed images was composed using three profiles (pre-treatment, post-camouflage, and post-orthognathic surgery) and six nose types. These photographs were shown to the three groups (orthodontists, plastic surgeons, and lay people), and they were asked to assign an attractiveness score to each photo ranging from 0 to 100, with 0 indicating the least attractive and 100 indicating the most attractive.

**Results:**

For the convex nose profiles, anterior movement of the mandible obtained by orthognathic surgery did not result in a significant change in the scores given by the lay people. When surgical or camouflage treatment was not implemented and, instead, just rhinoplasty was performed for these profiles, there was a significant increase in the aesthetic scores given by all groups. For the straight nose profiles, orthognathic surgery increased the attractiveness scores given by all groups. Furthermore, for all the profiles, extraction treatment did not affect the aesthetic scores given by any of the groups (*P* > 0.05).

**Conclusions:**

The lay people perceived that having a convex-bridged nose was a bigger problem than having a retrognathic profile. Overall, in terms of skeletal and dental orthodontic treatments, nose shape should be considered during the treatment planning process.

## Introduction

The lack of growth potential in adult skeletal class II patients makes it impossible to perform growth modification treatments like functional jaw orthopaedics. Out of all the available treatments for these patients, the first option is usually skeletal structure intervention in conjunction with orthodontics and surgery, which is referred to as orthognathic surgery. The other option involves masking the underlying skeletal problem by moving the maxillary dentoalveolar structures and correcting occlusion, which is a method called camouflage treatment [1].

Although orthognathic surgery techniques have advanced considerably and are now less traumatic than in the past, it may still be difficult to convince patients and their parents that they should undergo orthognathic surgery. When this is the case, the best way to achieve the desired results is performing camouflage treatment. Despite the fact that the treatment plan is easily established in severe skeletal class II adult patients, it is difficult to reach this decision in borderline cases. Several survey studies in the literature have evaluated the effects of both treatment options on facial aesthetics [1–6]; however, these studies concentrate on the effects on the jaws and lips (comprising the lower part of the face), while the effects of the nose and surrounding structures (comprising the middle third of the face) have been ignored.

The aims of the present study were to investigate the effects of different nose types on profile aesthetics obtained at the end of treatment options—camouflage and surgery—in skeletal class II borderline cases and to provide a prediction for orthodontists in treatment planning. Thus, while the treatment alternatives are explained to the patient, a more conscious orientation can be achieved and more aesthetic results can be obtained at the end of the treatment.

## Materials and methods

**Fig. 1:**
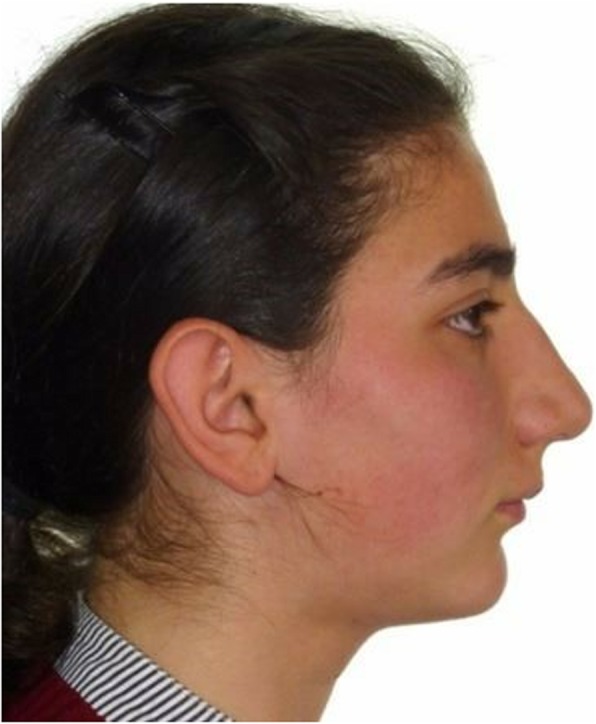
Reference photograph used in the present study

This study was approved by the clinical research ethics committee of Cumhuriyet University. Patients who presented with (A) halted growth status (cervical stage 6, according to the cervical vertebral maturation method), (B) a skeletal class II pattern caused by mandibular retrognathia, (C) a minimum ANB angle of 6°, (D) no previous history of orthodontic treatment, and (E) no striking elements on the face, such as asymmetry or scars, were identified from the department archive records. Three subjects who met the criteria were selected, and their resting profile photographs were shown to 16 orthodontists. They were then asked about their potential treatment plan choices (e.g. camouflage or surgery). Based on their answers, the patient who had the closest preference rate to each other between two treatment options was chosen, considering that it was a borderline case (pre-treatment, PT) (Fig. 1). Informed consent of the subject was obtained.

## Creating constructed photographs

**Fig. 2:**
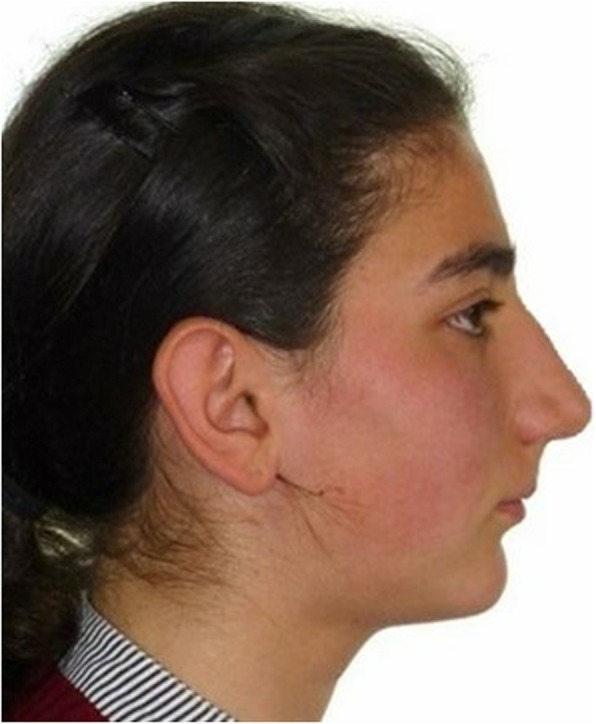
Constructed photograph after virtual camouflage treatment

In the profile photo of the selected case, who presented with an 8-mm pre-treatment overjet, the extraction of the upper first premolars and the retraction of the upper incisors were simulated with Dolphin imaging software (version 11.8, Dolphin Imaging, Chatsworth, CA). It was observed that incisor retraction, which is required to reach the normal overjet amount (2 mm), caused a 3–3.5-mm upper lip retraction relative to the E plane. This constructed photograph was used as a profile image for performing camouflage treatment (CT) (Fig. 2).

**Fig. 3:**
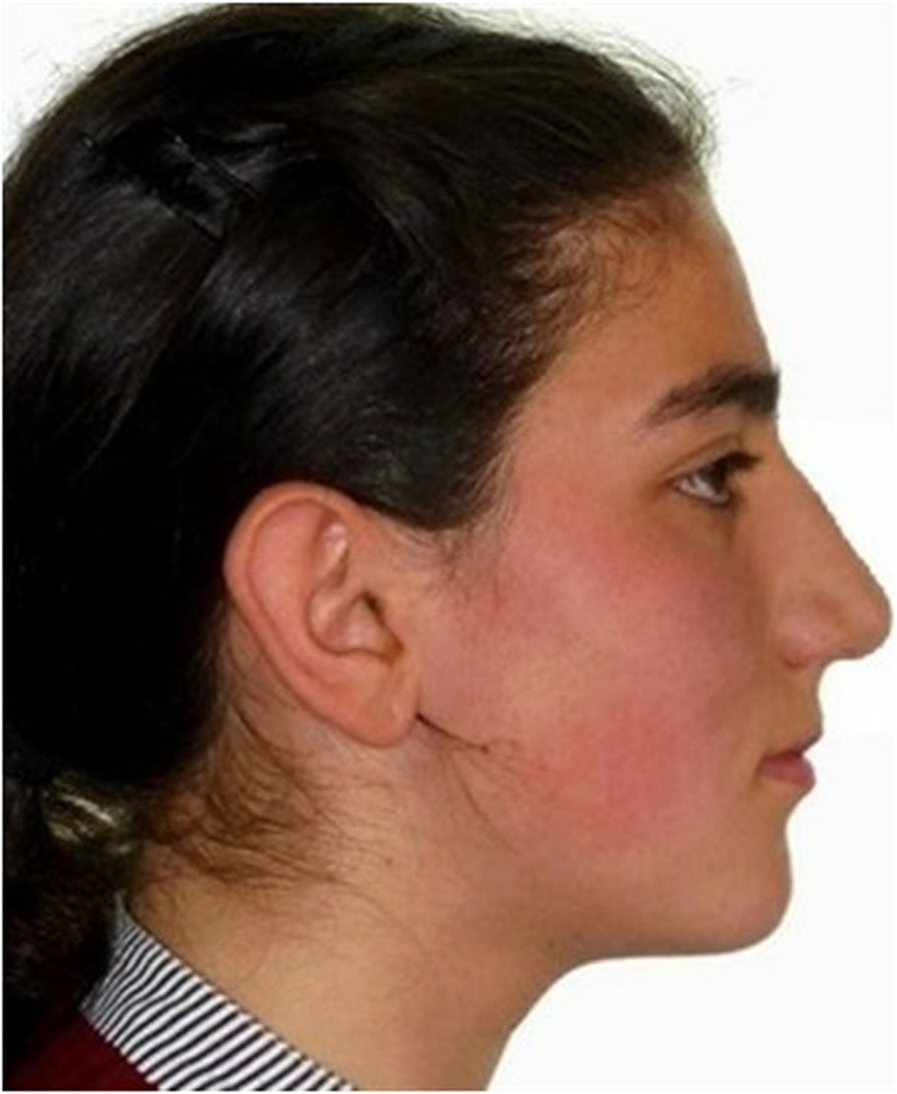
Constructed photograph after virtual orthognathic surgery

Using the same software, mandibular advancement surgery was simulated in such a way that the angle of the ANB would be 2° and the overjet would be 2 mm. Constructed in this way, the photograph was used as a profile image for performing orthognathic surgery (represented by OS in the figures) (Fig. 3).

**Fig. 4:**
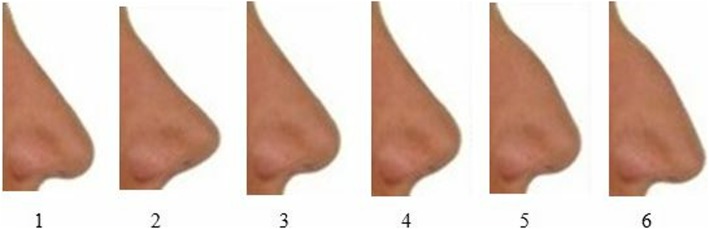
Nose types used in the present study

Six of the nose types mentioned in the book *Functional Reconstructive Nasal Surgery* written by Huizing and Groot were selected (Fig. 4) [7]. These nose types were adapted on the three profile photos (e.g. PT, CT, and OS) using the facetouchup simulation program (Pixineers Inc., Calgary, AB, Canada), which allows for virtual plastic surgery.

**Fig. 5:**
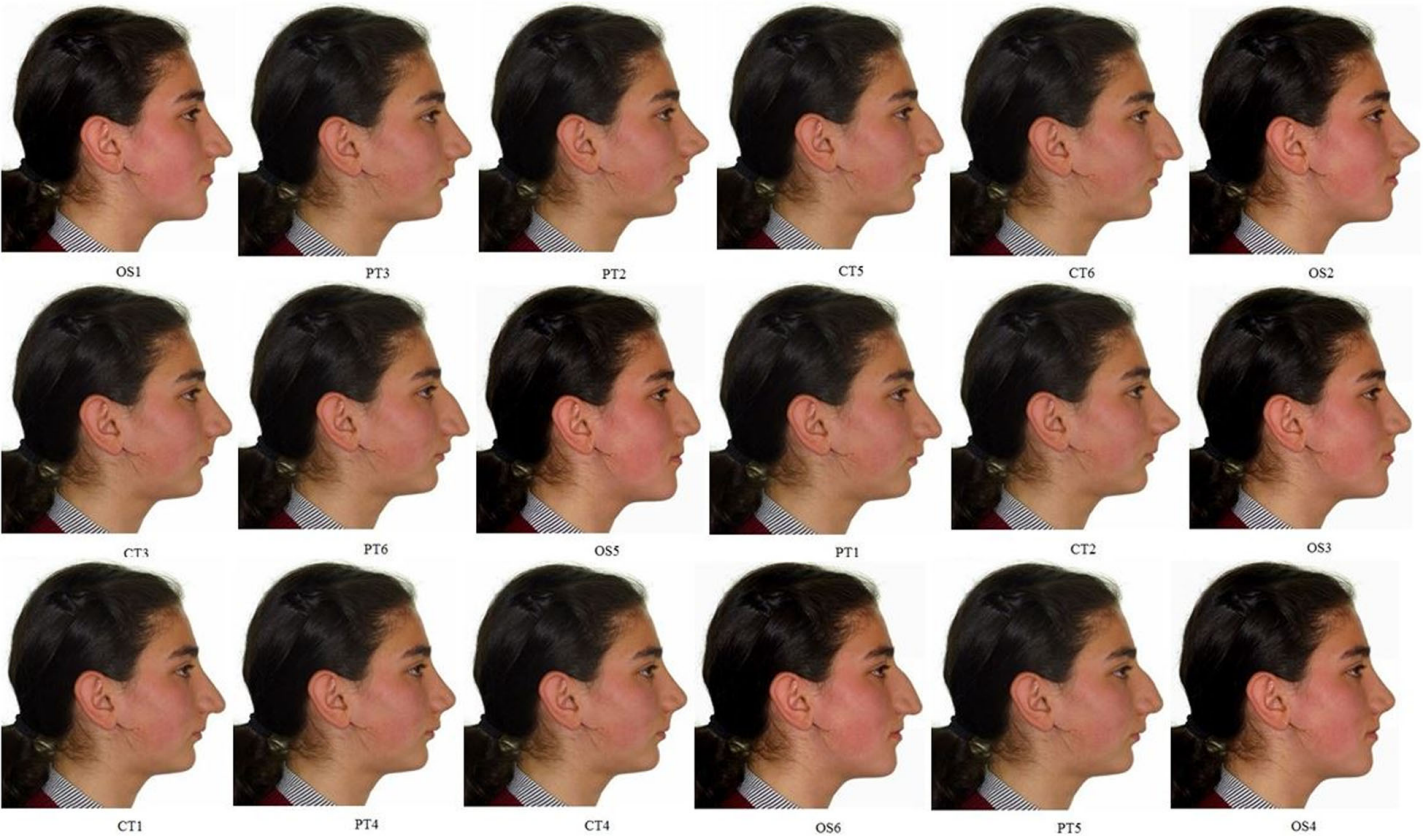
The final version of the survey

The 18 constructed profile photographs, which were randomly ordered without any grouping or ranking, were shown to three groups: 31 orthodontists, 34 plastic surgeons, and 34 lay people. They were asked to assign an aesthetic score to each photo ranging from 0 to 100, with 0 indicating the least attractive and 100 indicating the most attractive (Fig. 5). Participants were not subjected to orientation to allow for their focusing specifically on the nose profile, and they were asked to score randomly.

## Statistical analysis

At the beginning of this study, it was detected that a sample size of 99 participants—consisting of 31 orthodontists, 34 plastic surgeons, and 34 lay people—would yield a power of .916 with a nondirectional alpha risk of .05. The Mann-Whitney *U*, Kruskal-Wallis, Friedman, Wilcoxon, and Pearson’s correlation coefficient tests were used for the statistical analysis, and *P* values of less than .05 were considered statistically significant. Statistical analysis was performed using the Statistical Package for the Social Sciences (IBM SPSS Statistics for Windows, Version 22.0. Armonk, NY).

## Results

The highest and lowest scores were given to the profiles OS4 and CT6, respectively, by all the groups (Table 1).

**Table 1:** Attractiveness ranking of photographs according to the groups

Ranking of attractiveness	Orthodontist (mean ± standard deviation)	Plastic surgeon (mean ± standard deviation)	Lay persons (mean ± standard deviation)
1	OS4 (83.23 ± 15.89)	OS4 (78.97 ± 12.54)	OS4 (80.88 ± 13.62)
2	OS3 (80.97 ± 13.93)	OS3 (78.53 ± 14.27)	OS3 (79.50 ± 23.71)
3	OS1 (63.16 ± 14.73)	OS2 (67.79 ± 17.67)	CT3 (62.88 ± 19.98)
4	OS2 (62.68 ± 14.87)	CT3 (64.26 ± 16.42)	OS2 (60.88 ± 26.06)
5	PT4 (60.14 ± 17.02)	PT4 (62.85 ± 15.78)	PT4 (60.74 ± 21.03)
6	PT3 (58.32 ± 13.13)	OS1 (62.35 ± 14.62)	OS1 (59.91 ± 23.93)
7	CT3 (56.13 ± 14.35)	PT3 (62.35 ± 13.77)	CT4 (57.21 ± 24.65)
8	CT4 (55.81 ± 15.22)	CT4 (59.68 ± 16.03)	PT3 (56.62 ± 24.04)
9	PT1 (50.97 ± 16.45)	PT1 (55.09 ± 14.06)	CT1 (50.29 ± 25.16)
10	OS5 (50.97 ± 14.45)	CT2 (53.91 ± 17.73)	PT2 (47.79 ± 24.47)
11	CT1 (50.90 ± 15.40)	CT1 (53.74 ± 16.04)	PT1 (45.68 ± 26.11)
12	CT2 (46.13 ± 14.58)	PT2 (50.74 ± 20.23)	CT2 (42.65 ± 25.05)
13	OS6 (43.29 ± 17.31)	OS5 (49.26 ± 17.01)	OS5 (29.94 ± 20.11)
14	PT2 (43.06 ± 12.95)	OS6 (44.06 ± 16.63)	PT5 (26.91 ± 24.46)
15	PT5 (40.81 ± 15.65)	PT5 (42.29 ± 15.64)	OS6 (26.18 ± 21.07)
16	CT5 (37.26 ± 12.09)	PT6 (37.74 ± 15.52)	PT6 (25.15 ± 18.23)
17	PT6 (36.71 ± 12.24)	CT5 (37.06 ± 16.79)	CT5 (24.44 ± 16.86)
18	CT6 (31.13 ± 14.06)	CT6 (36.76 ± 14.86)	CT6 (20.47 ± 14.84)

*PT* pre-treatment, *CT* camouflage treatment, *OS* (orthognathic surgery)

When the scores provided by the three groups were compared, the observed differences were not found to be significant for the profile photos PT1, PT2, PT3, PT4, CT1, CT2, CT3, CT4, OS1, OS2, OS3, and OS4; however, there were statistically significant differences among the profile photos PT5, PT6, CT5, CT6, OS5, and OS6 (Table 2).

**Table 2:** Comparison of attractiveness scores among the groups

	Mean ± standard deviation (orthodontist)	Mean ± standard deviation (plastic surgeon)	Mean ± standard deviation (lay persons)	*P*
PT1	50.97 ± 16.45	55.09 ± 14.06	45.68 ± 26.11	.131
PT2	43.06 ± 12.95	50.74 ± 20.23	47.79 ± 24.47	.262
PT3	58.32 ± 13.13	62.35 ± 13.77	56.62 ± 24.04	.410
PT4	60.14 ± 17.02	62.85 ± 15.78	60.74 ± 21.03	.746
PT5	40.81 ± 15.65^a^	42.29 ± 15.64^a^	26.91 ± 24.46^b^	.001*
PT6	36.71 ± 12.24^a^	37.74 ± 15.52^a^	25.15 ± 18.23^b^	.001*
CT1	50.90 ± 15.40	53.74 ± 16.04	50.29 ± 25.16	.591
CT2	46.13 ± 14.58	53.91 ± 17.73	42.65 ± 25.05	.129
CT3	56.13 ± 14.35	64.26 ± 16.42	62.88 ± 19.98	.052
CT4	55.81 ± 15.22	59.68 ± 16.03	57.21 ± 24.65	.502
CT5	37.26 ± 12.09^a^	37.06 ± 16.79^a^	24.44 ± 16.86^b^	.001*
CT6	31.13 ± 14.06^a^	36.76 ± 14.86^a^	20.47 ± 14.84^b^	.001*
OS1	63.16 ± 14.73	62.35 ± 14.62	59.91 ± 23.93	.497
OS2	62.68 ± 14.87	67.79 ± 17.67	60.88 ± 26.06	.446
OS3	80.97 ± 13.93	78.53 ± 14.27	79.50 ± 23.71	.143
OS4	83.23 ± 15.89	78.97 ± 12.54	80.88 ± 13.62	.242
OS5	50.97 ± 14.45^a^	49.26 ± 17.01^a^	29.94 ± 20.11^b^	.001*
OS6	43.29 ± 17.31^a^	44.06 ± 16.63^a^	26.18 ± 21.07^b^	.001*

*PT* pre-treatment, *CT* camouflage treatment, OS orthognathic surgery

*Significant at *P* < 0.05 by Kruskal-Wallis test

^a,b^Groups with the same letter are not significantly different (Mann-Whitney *U* test)

However, statistically significant and strong correlations were observed for all the binary group matches (Table 3).

**Table 3:** Evaluation of correlations among the groups

	Orthodontist	Plastic surgeon	Lay persons
Orthodontist			
*r*	1	.97	.91
*p*		.001*	.001*
Plastic surgeon			
*r*	.97	1	.93
*p*	.001*		.001*

*Significant at *P* < 0.05 by Pearson’s correlation coefficient test

For the first four nose types, the differences between pre-treatment and orthognathic surgery as well as camouflage and orthognathic surgery were found to be significant within all three groups, while the differences between pre-treatment and camouflage were not found to be significant. For the fifth and sixth nose types, the differences between pre-treatment and orthognathic surgery as well as camouflage and orthognathic surgery were found to be significant within both the orthodontist and plastic surgeon groups, while the differences between pre-treatment and camouflage were not found to be significant. However, there were no significant differences between the three profile images within the lay people group (Table 4).

**Table 4:** Intra-group comparison of profiles according to nose types

	Mean ± standard deviation (orthodontist)	Mean ± standard deviation (plastic surgeon)	Mean ± standard deviation (lay persons)
PT1	50.97 ± 16.45^a^	55.09 ± 14.06^a^	45.68 ± 26.11^a^
CT1	50.90 ± 15.40^a^	53.74 ± 16.04^a^	50.29 ± 25.16^a^
OS1	63.16 ± 14.73^b^	62.35 ± 14.62^b^	59.91 ± 23.93^b^
*P*	.001*	.001*	.001*
PT2	43.06 ± 12.95^a^	50.74 ± 20.23^a^	47.79 ± 24.47^a^
CT2	46.13 ± 14.58^a^	53.91 ± 17.73^a^	42.65 ± 25.05^a^
OS2	62.68 ± 14.87^b^	67.79 ± 17.67^b^	60.88 ± 26.06^b^
*P*	.001*	.001*	.001*
PT3	58.32 ± 13.13^a^	62.35 ± 13.77^a^	56.62 ± 24.04^a^
CT3	56.13 ± 14.35^a^	64.26 ± 16.42^a^	62.88 ± 19.98^a^
OS3	80.97 ± 13.93^b^	78.53 ± 14.27^b^	79.50 ± 23.71^b^
*P*	.001*	.001*	.001*
PT4	60.14 ± 17.02^a^	62.85 ± 15.78^a^	60.74 ± 21.03^a^
CT4	55.81 ± 15.22^a^	59.68 ± 16.03^a^	57.21 ± 24.65^a^
OS4	83.23 ± 15.89^b^	78.97 ± 12.54^b^	80.88 ± 13.62^b^
*P*	.001*	.001*	.001*
PT5	40.81 ± 15.65^a^	42.29 ± 15.64^a^	26.91 ± 24.46^a^
CT5	37.26 ± 12.09^a^	37.06 ± 16.79^a^	24.44 ± 16.86^a^
OS5	50.97 ± 14.45^b^	49.26 ± 17.01^b^	29.94 ± 20.11^a^
*P*	.001*	.001*	.055
PT6	36.71 ± 12.24^a^	37.74 ± 15.52^a^	25.15 ± 18.23^a^
CT6	31.13 ± 14.06^a^	36.76 ± 14.86^a^	20.47 ± 14.84^a^
OS6	43.29 ± 17.31^b^	44.06 ± 16.63^b^	26.18 ± 21.07^a^
*P*	.001*	.001*	.054

*PT* pre-treatment, *CT* camouflage treatment, *OS* orthognathic surgery

*Significant at *P* < 0.05 by Friedman test

^a,b^Groups with the same letter are not significantly different (Wilcoxon test)

## Discussion

The literature presents some descriptive data about the borderline case for orthognathic surgery [8, 9]. Proffit et al. stated that, if an overjet is greater than 10 mm, it is likely orthognathic surgery will be needed in order to successfully correct the malocclusion [8]. In another study, an ANB angle of 6° was presented as a cutoff point where it can be consistently perceived that there is an improvement after surgery and to minimise the incidence of the profile worsening after treatment [9]. The patient in the present study presented with an ANB angle of 7° and an 8.5-mm overjet. In this respect, it was thought that these measurements were similar to the data defining the limits of orthognathic surgery, and, thus, it could be considered a borderline case. Additionally, a male or female subject should have been preferred for the study. Since it has been stated that females are more willing to have adult orthodontic treatment compared to males, a female subject was chosen for the present study [10]. The literature does not currently include any study examining the correlation between nose type and malocclusions; therefore, we were unable to identify the types of nose most common in class II subjects. The nose types which were presented as the most common abnormalities and anatomical variations in the society were used in this study [7].

Methods like photographs or silhouettes have been used in the literature to evaluate profile changes. Shelly et al. and O’Neill et al. utilised silhouettes to evaluate the contribution of mandibular orthognathic surgery and functional treatment [9, 11]. Silhouettes were chosen to standardise the images by avoiding factors that could affect perceptions of attractiveness, such as hairstyles, make-up, or clothing. However, various computer programmes have made standardisation possible in studies that use photographs [12, 13], and, therefore, photographs were used in the present study. Additionally, it was noted that the most commonly used measurement methods in the survey studies were the Likert scale and visual analogue scale (VAS) [9, 11–13]. Due to advantages like being highly sensitive to changes and easy to use, the VAS was selected as the preferred method of measurement for the present study.

In this study, when the aesthetic scores given by each group were compared, it was observed that the orthodontists and plastic surgeons assigned very similar scores. However, lay people’s scores differed from the professional groups for PT5, PT6, CT5, CT6, OS5, and OS6 (Table 2). Regardless of profile type, lay people assigned lower scores to the profiles with the fifth and sixth noses (e.g. convex noses) than the other groups. This can be explained by the fact that society attaches greater importance to the nose and often rejects the convex nose types, even if it is positioned in a class I skeletal pattern. It has already been reported that rhinoplasty is most commonly applied to these nasal types [14]. In addition, it was observed that the standard deviation for the lay people group was higher than that of the other two groups. In a similar study, standard deviations of scores from lay people were higher, and Tsang et al. noted that lay people were less aware of changes resulting from orthognathic surgery than the professional groups [15]. In their study, Burcal et al. reported that lay people experienced difficulties in noticing jaw movements less than 4 mm and that the ability to perceive change was greater in dental professionals [16].

Even though different results were obtained in regard to the six photographs, there were high correlations found between the groups in terms of scores; specifically, correlation coefficients of .97 (orthodontists and plastic surgeons), .91 (orthodontists and lay people), and .93 (plastic surgeons and lay people) were found in the present study (Table 3). It was revealed that perceptions of attractiveness were very similar among all three groups. In other words, there was a common trend among individuals in terms of aesthetic evaluation regardless of education level or clinical training.

When the results were examined in terms of nasal type, it was seen that the results were different according to the straight (1, 2, 3, and 4) and convex (5 and 6) nose types (Table 4). For the professional groups, while the differences between OS–PT and OS–CT were statistically significant for the profiles exhibiting the fifth and sixth nose types with a convex bridge, the difference between CT and PT was found to be insignificant. In the lay people group, the differences were found insignificant for all groups (e.g. OS–PT, OS–CT, and PT–CT). As can be seen from these findings, if an adult patient with a convex nose bridge does not choose the orthognathic surgery option, camouflage treatment will not result in a statistically significant negative result, as determined by both professionals and lay people.

When orthognathic surgery was preferred for convex nose profiles, a better image was obtained for the professionals, but no significant improvement was achieved for the lay people. For instance, when orthognathic surgery was performed for PT5 (PT5→OS5), the mean scores given by the groups changed as follows: orthodontists (40.81→50.97), plastic surgeons (42.29→49.26), and lay people (26.91→29.94) (Table 4). This suggests that, for lay people, having a convex bridged nose is a greater issue than having a class II profile or tooth-extracted profile. When a normal nose and mandible were provided for the same patient using rhinoplasty and orthognathic surgery (PT5→OS3), the mean scores markedly increased: orthodontists (40.81→80.97), plastic surgeons (42.29→78.53), and lay people (26.91→79.50) (Table 4). As shown, for convex nose types, orthognathic surgery without rhinoplasty will not satisfy patients’ expectations, especially when considering the risks, costs, and post-operation recovery period. This same assertion applies to camouflage treatment as well (PT5→CT5 versus PT5→CT3).

Another notable result was that, when just rhinoplasty without camouflage or orthognathic surgery was performed on a skeletal class II patient with a convex nasal type (PT5→PT3), there were severe increases in the scores of the orthodontists (40.81→58.32), plastic surgeons (42.29→62.35), and lay people (26.91→56.62) (Table 4). Regardless of which treatment option (e.g. camouflage or orthognathic surgery) was preferred, rhinoplasty should be considered as a part of the treatment plan for the patients with convex-bridged nose.

In terms of the profiles obtained with straight nose types (1, 2, 3, and 4), the differences between OS–PT and OS–CT were found to be statistically significant for all three groups, while the differences between CT–PT were insignificant. As can be seen from these findings, all three groups perceived orthognathic surgery, for patients with straight noses, as positively contributing to their profile aesthetics. Moreover, the groups agreed that camouflage therapy did not have a negative effect on the profiles of class II individuals with straight noses. In fact, the mean scores of some profiles with camouflage treatment were higher than those with pre-treatment (Table 4).

Currently, it is relatively easy to take photographs of individuals at any time via the widespread use of photo-taking mobile phones. Moreover, with this, the ability to instantly check facial appearances and share these photos to social media accounts has significantly increased society’s awareness of external appearances. Especially with the increase in the ‘selfie’ trend in recent years, the facial zone has become more significant than the overall external appearance; therefore, even though dental complaints are still more common, the number of complaints about facial aesthetics has been increasing with time. For this reason, in addition to establishing an ideal occlusion according to cephalometric analysis, orthodontists should share predictive digital photos, which represent the various treatment options with patients, by taking advantage of developing imaging systems.

As a limitation of this study, it can be stated that only profile photographs showing patients in a rest position were used. If frontal, 3/4 profile, or smiling photographs had also been used, the aesthetic perceptions of the participants could have changed. There are studies using these images in the literature [17, 18]. Another issue that should be emphasised is soft tissue changes as a result of ageing, which especially occur in the nose and lips. West and McNamara examined changes in the craniofacial complex from adolescence to middle adulthood [19] and reported that females exhibited nasal growth that progressed downward and forward, with a slight retrusion of the lips over time. Similarly, Behrents stated that the nose became more prominent with ageing [20]. Bishara et al. claimed that the upper and lower lips showed significantly more retrusion in relation to the aesthetic line between 15 and 45 years of age in both males and females [21]. Consequently, even if adolescence growth has been completed, with age, the nose will become more prominent and the lips will exhibit greater retrusion. This should be taken into consideration, especially when interpreting the present results on camouflage treatment.

## Conclusions

The conclusions of the present study are as follows:

- For all nose types, the highest scores were given by the study participants to the orthognathic surgery profiles; however, in terms of the convex nose profiles, there were no statistically significant differences found between the scores given by the lay people (PT–CT, PT–OS, and CT–OS).
- When differences between the groups were evaluated, none were found between the scores provided by the orthodontists and plastic surgeons; however, statistically significant differences were detected between the lay people and the two professional groups in terms of their evaluation of the convex nosed profiles (PT5, PT6, CT5, CT6, OS5, and OS6).
- Lay people perceived that having a convex-bridged nose was a bigger problem than having a retrognathic profile. Straight-bridged noses were found to be more acceptable by all groups.
- When compared to the pre-treatment profiles, camouflage treatment did not produce a statistically significant effect on the aesthetic scores for all nose types.

## Data Availability

The datasets used and analysed during the current study are available from the corresponding author on reasonable request.

## Abbreviations

CT: Camouflage treatment
OS: Orthognathic surgery
PT: Pre-treatment
VAS: Visual analogue scale
